# A 1.8–2.7 GHz Triple-Band Low Noise Amplifier with 31.5 dB Dynamic Range of Power Gain and Adaptive Power Consumption for LTE Application

**DOI:** 10.3390/s22114039

**Published:** 2022-05-26

**Authors:** S. Ali Hosseini Asl, Reza E. Rad, Behnam S. Rikan, YoungGun Pu, Keum Cheol Hwang, Youngoo Yang, Kang-Yoon Lee

**Affiliations:** 1 Department of Electrical and Computer Engineering, Sungkyunkwan University, Suwon 16419, Korea; saha@skku.edu (S.A.H.A.); reza@skku.edu (R.E.R.); behnam@skku.edu (B.S.R.); hara1015@skku.edu (Y.P.); khwang@skku.edu (K.C.H.); yang09@skku.edu (Y.Y.); 2 SKAIChips Co., Ltd., Suwon 16419, Korea

**Keywords:** LTE, wideband, low NF, SOI, RF front-end LNA, wireless communication

## Abstract

This paper presents a multi-gain radio frequency (RF) front-end low noise amplifier (LNA) utilizing a multi-core based on the source degeneration topology. The LNA can cover a wide range of input and output frequency matching by using a receiver (RX) switch at the input and a capacitor bank at the output of the LNA. In the proposed architecture here, to avoid the saturation of RX chain, 12 gain steps including positive, 0 dB, and negative power gains are controlled by a mobile industry processor interface (MIPI). The multi-core architecture offers the ability to control the power consumption over different gain steps. In order to avoid the phase discontinuity, the negative gain steps are provided using an active amplification and T-type attenuation path that keeps the phase discontinuity below ±5 degrees between two adjacent power gain steps. Using the multi-core structure, the power consumption is optimized in different power gains. The structure is enhanced with the adaptive variable cores and reactance parameters to maintain different power consumption for different gain steps and remain the output matching in an acceptable operating range. Furthermore, auxiliary linearization circuitries are added to improve the input third intercept point (IIP3) performance of the LNA. The chip is fabricated in 65 nm complementary metal-oxide semiconductor (CMOS) silicon on insulator (SOI) process and the die area is 0.308 mm^2^. The proposed architecture achieves the IIP3 performance of −10.2 dBm and 8.6 dBm in the highest and lowest power gains, which are 20.5 dB and −11 dB, respectively. It offers the noise figure (NF) performance of 1.15 dB in the highest power gain while it reaches 14 dB when the power gain is −11 dB. The LNA consumes 16.8 mA and 1.33 mA current from a 1 V power supply that is provided by an on-chip low-dropout (LDO) when it operates at the highest and lowest gains, respectively.

## 1. Introduction

The growing demands for high-speed network communications and the development of frequency bands in new mobile communication devices, such as the long-term evolution (LTE) and fifth-generation (5G), lead to radio frequency (RF) front-end low noise amplifiers (LNAs) being complicated [1]. Considering advancements in a new generation of mobile communication, still, the previous standards and frequency bands are applicable. Thus, mobile devices are still required to support the previous generations; meanwhile, when a new generation is presented (e.g., the sixth generation), the previous generations include their frequency band in new devices [2]. Hence, it demands improvements both in performance and at the architectural level. In addition, all the new ideas are applicable to other bands of interest. Therefore, receivers (RXs) need to support different frequency bands [3]. Nonetheless, lower power consumption to increase the battery life of mobile devices has always been one of the most important concerns in RF transceivers design. Thereby, it is inevitable to present new techniques to meet the new concepts for LNAs by retaining RF performances.

One of the other essential issues in portable communication devices is the quality of signals between the signal sources and users. Since the distance of RXs and transceivers (TXs) cannot be fixed, the received power level of a signal is variable at the antenna. Thus, operating in a large dynamic range is the obvious demanding characteristic of LNAs. When a portable device is far enough from a signal source (e.g., when the cellphone is used in the basement of a building) and the received signal at the RX antenna is very small [4], the LNA has to amplify the signal with maximum gain and minimum noise figure (NF) to appear a satisfactory signal-to-noise ratio (SNR) at the output of RX. In another scenario, if the received signal is so large at the antenna and the LNA would operate with the maximum power gain, the delivered signal to subsequent stages is destroyed due to the non-linearity of LNA [3]. Hence, the signal has to be attenuated. Although the NF performance is degraded, the SNR of the received signal is high and the degradation of NF performance could be negligible and the power gain could be relaxed.

In [5], low and high gain steps are provided by a parallel reactance with the load inductor, which is provided using a transistor while capacitor banks are used to provide fine gain tunings. The proposed structure [5] provides a limited number of gain steps and the area occupation of the capacitors is relatively large for the fine gain tuning operation. Meanwhile, such a structure results in a higher NF demand in LTE application.

Phase discontinuity is the other concern in the design of the RX system. If the end of a waveform and the beginning of the next repetition, during switching to the subsequent gain steps, are not in the same phase it can, on occasion, cause periodic spectral regrowth and distortion. Due to the high-frequency spectral regrowth that is caused by the abrupt phase changes of the repetitions, phase discontinuity can cause a periodic spectral regrowth that results in an undesirable distortion. Additionally, phase discontinuity produces a huge increase in the components of distortion in comparison with the normal line spectra representative of a single sinewave when the sinewave samples are being played back. Given that, the abrupt phase changes occur in gain switching, therefore the difference in phase discontinuity of each gain step must be below ±5 degrees compared to its higher power gain step or lower power gain step. Accordingly, to achieve 0 dB power gain or negative power gain steps, a passive path is not recommended [6].

The works in [1,7] use the conventional cascode structure and source degeneration technique while achieving a single power gain of 20–22 dB with low NF performance and there are no addition gain modes to avoid the saturation of Rx. A structure with four gain steps where the negative gain steps are generated using a bypass mode is presented in [3]. Using the bypath path causes the problem of phase discontinuity, which distorts the signal and is not desirable, especially when the communication is based on phase modulation.

This paper is organized as follows; Section 2 introduces the design of the overall architecture. In Section 3, the multi-core description is given. In Section 4, the experimental performances are summarized, and Section 5 presents the conclusions of the letter.

## 2. Design of Overall Architecture

**Figure 1:**
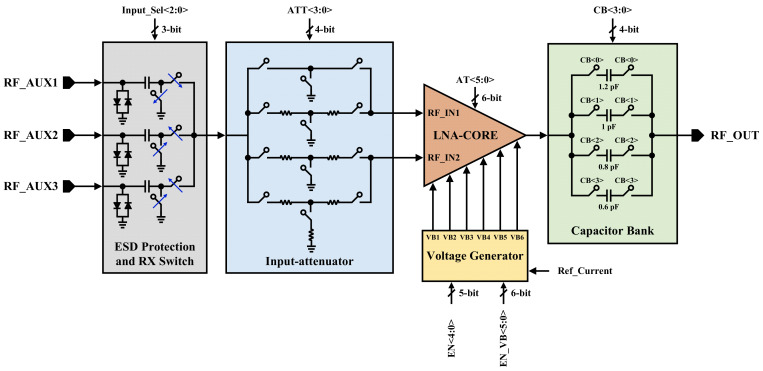
Top block diagram of proposed RF front-end.

The overall architecture of the proposed RF front-end consists of an input ESD protection and triple-band RX switch at the input, input-attenuator, LNA-core, voltage generator, and capacitor bank at the output, as stated in Figure 1.

The proposed architecture is enhanced to provide a total of 12 gain steps for two separate commercial standards. In fact, the proposed structure operates in two modes to cover two different markets. Mode0 includes seven gain steps (G0x–G6x) and six gain steps (G0–G5) are designed for Mode1, it should be noted that G0x and G0 have the same performance in both modes. Thus, the complexity of the structure is increased to cover a wide frequency range from 1.8 GHz to 2.7 GHz and provide a total of 12 power gain steps in two different modes. 

The input RX switch splits the frequency band coverage into three different zones. The input-attenuator unit is located between the RX switch and LNA-core to provide bypass or attenuation operation for the RF inputs of the LNA-core. The reconfigurable LNA-core performs the low noise amplification over the received RF inputs from the input-attenuator. To provide an adaptive power consumption for 12 gain steps, the digitally controlled voltage generator is placed to adjust the current consumption of the LNA-core corresponding to every power gain step. The output capacitor bank is providing adaptive output matching for all of the frequency bands and gain steps, while keeping the phase variations below ±5 degrees between two adjacent power gain steps.

The triple-band structure is obtained through the input RX switches, hence the operating frequency bands for the three different auxiliary inputs would be switchable [8]. Accordingly, RF_AUX1, RF_AUX2, and RF_AUX3, cover 1.8–2 GHz, 2–2.4 GHz, and 2.4–2.7 GHz bands, respectively. The frequency bands of 1.8–2 GHz, 2–2.4 GHz, and 2.4–2.7 GHz are nominated as low-band (LB), mid-band (MB), and high-band (HB), respectively. LB, MB, and HB cover frequency of 200 MHz, 400 MHz, and 300 MHz bandwidths, respectively. To eliminate inputs affection, while one of the inputs is connected to the RX chain, the other inputs are connected to the GND (0 V). 8.2 nH, 6.2 nH, and 4.3 nH of high-quality series inductors (Murata LQP02HQ) are employed to match the input to the required frequency for LB, MB, and HB, respectively. According to the desired power gain, a mobile industry processor interface (MIPI) is programmed to select which path through the input-attenuator must be used. An adaptive biasing technique is used to change the biasing voltage due to the selected power gain. In the desired power gain, the voltage generator provides the required biasing voltages (VB1–VB6) to the LNA-core. The reconfigurable capacitor bank is implemented to cover the output matching (S22) for the operation in LB, MB, and HB to a 50-Ω load [9,10].

**Figure 2:**
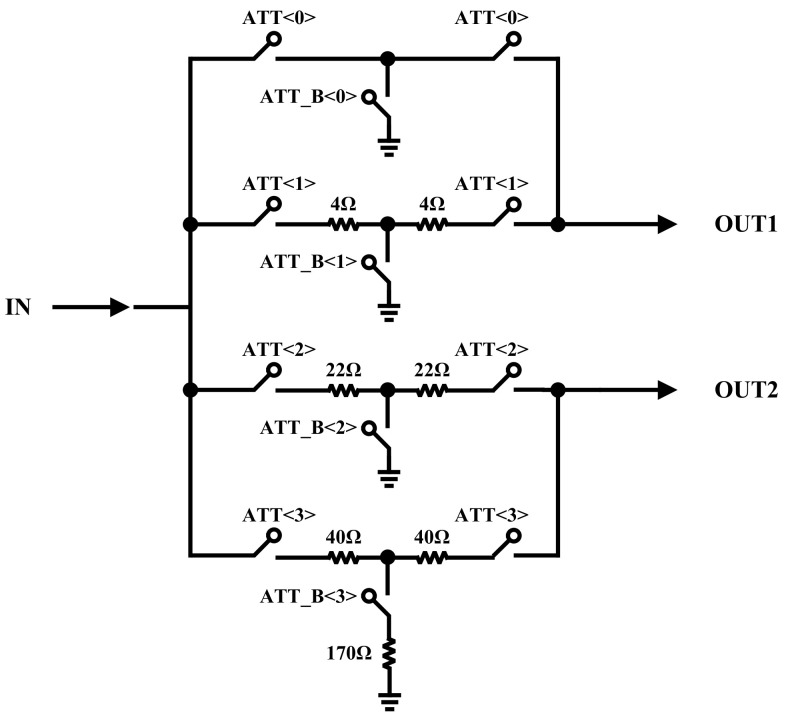
Block diagram of proposed input-attenuator.

Figure 2 demonstrates the proposed input-attenuator circuit where its outputs (OUT1 and OUT2) are connected to the inputs of the LNA-core. As mentioned in the Introduction, while the power and SNR of the received signal are low level, the input signal must be delivered directly to the LNA-core without any attenuation. Thus, the input signal passes through a switch and appears at the input of the LNA-core. In the other cases, depending on the power level and SNR of the input signal, the MIPI decides which path through the input-attenuator must be selected. Table 1 indicates the programmable digital control bits of the input-attenuator for all gain steps. It is worth mentioning that the input-attenuators could improve the input third intercept point (IIP3) performance.

The reconfigurable capacitor bank is located at the last stage to satisfy matching to 50-Ω output impedance. 0.6 pF, 0.8 pF, 1 pF, and 1.2 pF are the implemented capacitors in the capacitor bank, as shown in Figure 1. Table 2 demonstrated the programmable digital control bits of the capacitor bank for LB, MB, and HB.

## 3. Multi-Core and Voltage Generator Structure

**Figure 3:**
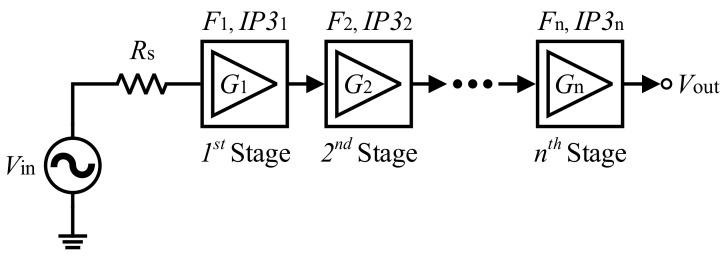
Block diagram of an n-stage cascaded system.

Figure 3 depicts an n-stage cascaded system block diagram. By Friis formula, the total noise contribution of every stage could be defined by the following expression [3]:

(1) $$F_{tot}=F_{1}+\frac{F_{2}-1}{G_{1}}+\frac{F_{3}-1}{G_{1}G_{2}}+\ldots +\frac{F_{n}-1}{G_{1}G_{2}\ldots \;G_{n-1}}$$

The noise of every stage is divided by the gain of previous stages, as derived in Equation (1). Hence the total noise in the RX chain would be generally written as:

(2) $$F_{tot}=F_{LNA}+\frac{F_{after\;LNA}-1}{G_{LNA}}$$

The maximum power gain of LNA is able to suppress the noise coming from subsequence stages, therefore the NF of LNA directly appears in the total noise factor of the RX chain. To achieve the best NF performance, it is evident that a high gain topology should be selected. Low NF performance, good matching to 50-Ω input impedance for narrowband applications, and high power performance are the attractive characteristics of the common-source (CS) stage with inductive degeneration structure [11]. The NF at the input resonance frequency and matched input for CS with degeneration inductor is given by the following expression [4,12]:

(3) $$F=1+g_{m}R_{s}\gamma {\left(\frac{\omega }{\omega_{t}}\right)}^{2}$$

**Figure 4:**
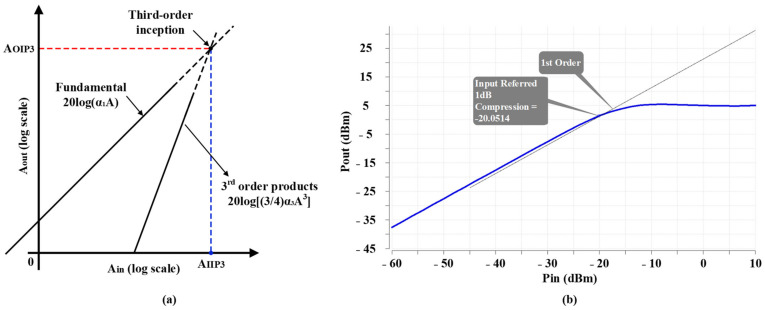
(**a**) Fundamental and third-order products amplitudes, and (**b**) post-layout simulation result of $P_{1\mathrm{dB}}$ performance at 1.8 GHz in LB for highest power gain step (G0x/G0).

The other important feature of LNAs is linearity. Figure 4a indicates a log-log scale of fundamental and third-order inception amplitudes. The IIP3 amplitude ($A_{IIP3}$) is defined at an input level where the fundamental and third-order inception amplitudes are met. Hence, to determine the IIP3 amplitude ($A_{IIP3}$), it can be written [4]:

(4) $$\left|\alpha_{1}A_{IIP3}\right|=\left|\frac{3}{4}\alpha_{3}A_{IIP3}^{3}\right|$$

(5) $$A_{IIP3}=\sqrt{\frac{3}{4}\left|\frac{\alpha_{1}}{\alpha_{3}}\right|}$$

(6) $$\frac{A_{IIP3}}{A_{1dB}}=\sqrt{\frac{4}{0.435}}\approx 9.6\;\mathrm{dB}$$

Therefore, the IIP3 can be obtained by following equation:

(7) $$IIP3=P_{1\mathrm{dB}}+9.6\;\mathrm{dBm}$$

Post-layout simulation result of $P_{1\mathrm{dB}}$ at 1.8 GHz in LB for G0x is indicated in Figure 4b. Following Equation (7), −10.45 dBm of IIP3 is achieved at 1.8 GHz in LB for the highest power gain step (G0x/G0).

**Figure 5:**
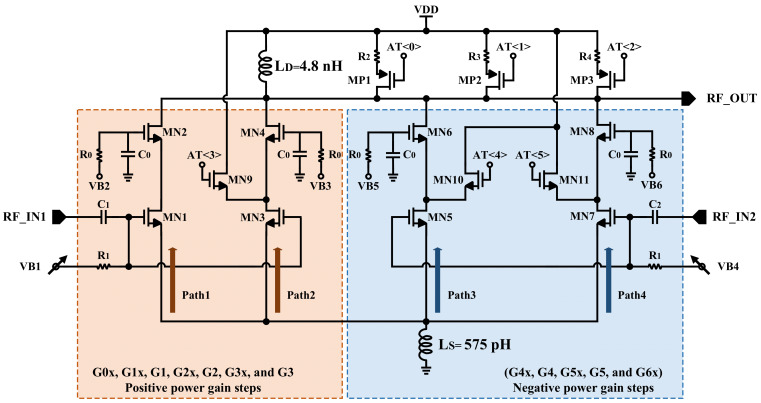
Multi-core structure common-source input stage with shared source degeneration inductor.

Figure 5 illustrates the proposed multi-core structure where the positive and negative gain steps are generally indicated. As demonstrated in Figure 5 the load switching (MP1, MP2, and MP3) and additional cascode (MN9, MN10, MN11) devices structures are applied to attenuate the input signal [4] for the required power gain steps. Additionally, the IIP3 performance is improved due to the attenuation [4].

The highest power gain (G0x/G0) is obtained while path1 and path2 are participated to amplify the input signal; in other words, VB1, VB2, and VB3 are biased to provide the required biasing voltage for operating transistors MN1, MN2, MN3, and MN4 in the saturation region. In this case, all other transistors are in the cutoff region (VB4, VB5, and VB6 are connected to GND). It is worth noting that if the biasing voltage of the cascode stage of a path connects to GND, the path could not participate in amplification.

To decrease the power consumption in power gain G1x from Mode0 and power gain G1 from Mode1, VB1 is biased in lower DC voltage level, path1 and path2 amplify the input signal. Since the SNR of the input signal is not high enough, to attenuate and improve IIP3 performance, the load switching and additional cascode devices structures are recommended.

In the other scenario, the lowest power gain (G6X) is achieved when the path4 is applied to cooperate in amplification; MN7 and MN8 operate in saturation region by biased voltage VB4 and VB6. In this case, to attenuate the input signal, the load switching (MP1, MP2, and MP3) and additional cascode (MN11) devices are employed. Table 3 indicated all the programmable digital control bits of the load switching and additional cascode devices for all power gain steps, from the highest power gain (G0x) to the lowest power gain (G6x) in Mode0 and from the highest power gain (G0) to the lowest power gain in Mode1, respectively.

**Figure 6:**
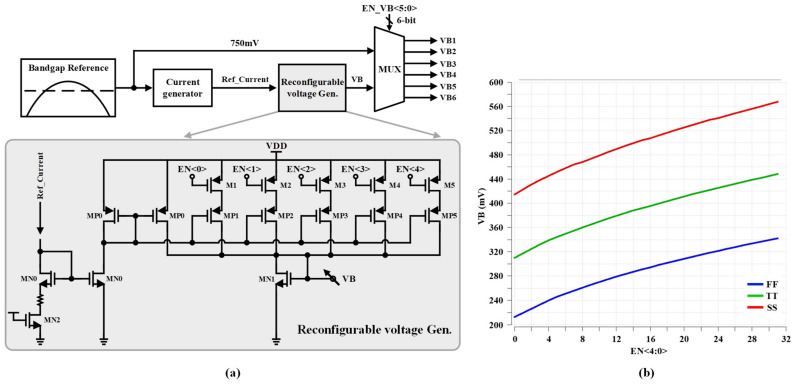
(**a**) Block diagram of the voltage generator, and (**b**) post-layout simulation results of the reconfigurable voltage Gen. in TT, SS, and FF corner conditions.

The voltage generator provides the required biasing voltages (VB1–VB6). To ensure that the LNA satisfies the RF specifications in PVT corner conditions as well, a low dropout (LDO) (1 V) supplies the current consumption of the LNA. The proposed reconfigurable voltage Gen. structure in [13] is implemented to generate a dynamic range of DC voltage by trimming EN<4:0>, as shown in Figure 6a. The designed reconfigurable voltage Gen. provides a range of 140 mV DC voltage from 310 mV to 450 mV to generate VB1 and VB4 voltages in typical-typical (TT) corner condition. In the fast-fast (FF) corner case, if the DC biasing voltage of the input stage (VB1 or VB4) of the CS topology remains as well as TT corner case, the current consumption will be increased. Therefore, in the FF corner case, the DC biasing voltage level of the input stage has to decrease; consequently, the current consumption will be reduced. Contrariwise, the power gain is reduced and S11 degraded in the slow-slow (SS) corner condition if the input stage bias voltage keeps the same value of voltage as well as TT corner case. Hence, to obtain the same RF performance, the DC biasing voltage level of the input stage has to increase.

The most interesting specification of the proposed reconfigurable voltage Gen. in [13] is that the generated biasing voltage varies intrinsically in corner conditions to remain within the RF specifications as well as TT condition. Figure 6b illustrates the post-layout simulation of the reconfigurable bias Gen. in TT, SS, and FF corner conditions. As shown in Figure 6b, generated biasing voltage is automatically increased and decreased in SS and FF corner conditions, respectively, to satisfy the RF specifications. The bandgap reference [14] provides the desired voltage reference (750 mV) for the current generator and the cascode stages of the LNA-core (VB2, VB3, VB5, and VB6). A multiplexer is employed to control the biasing voltages (VB1–VB6) of the LNA-core by the programmable digital control bits EN_VB<5:0>. Table 4 shows the biasing voltage values for all power gain steps in both Mode0 and Mode1. All implemented transistor sizes are indicated in Table 5.

## 4. Experimental Results

**Figure 7:**
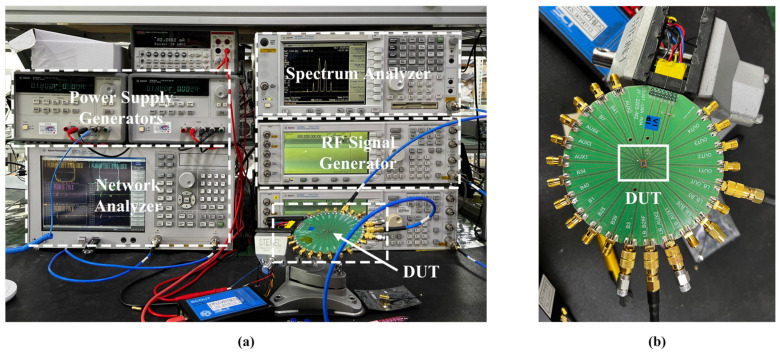
(**a**) Measurement environment, and (**b**) PCB and device under test of the proposed LNA.

**Figure 8:**
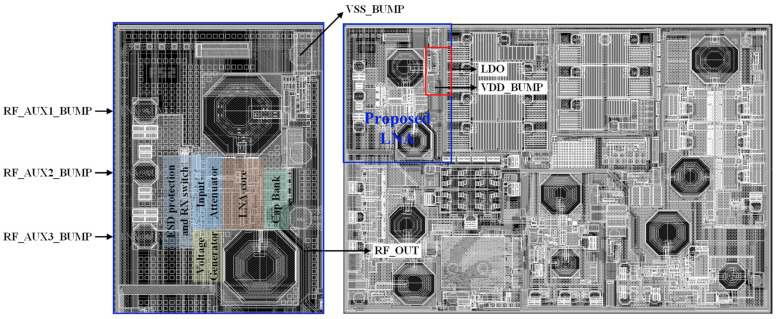
Top layout of the chip, proposed LNA and location of the ESD protection and RX switch at the input, attenuator, LNA-core, voltage generator, and capacitor bank.

The proposed triple-band, multi-gain RF front-end is implemented in a 65-nm complementary metal-oxide semiconductor (CMOS) silicon on insulator (SOI) process with an active die area of 0.308 mm^2^. The measurement environment is illustrated in Figure 7a, where power supply generators, spectrum analyzer, network analyzer, RF signal generator, and device under test (DUT) are applied for measurement. Figure 7b shows the print circuit board (PCB) and DUT. The top layout of chip, the proposed LNA, and location of the electrostatic discharge (ESD) protection and RX switch, input-attenuator, LNA-core, voltage generator, and capacitor bank are stated in Figure 8. The architecture provides a dynamic range of power gain from −11 dB to 20.5 dB. To measure the performance of the RF front-end, the supply voltage (1 V) is provided by an LDO. Table 6 presents the power consumption in all gain steps for Mode0 and Mode1. The power consumption is adapted according to the power gain from 1.33 mW (the lowest power gain) to 16.8 mW (the highest power gain), as shown in Table 6.

**Figure 9:**
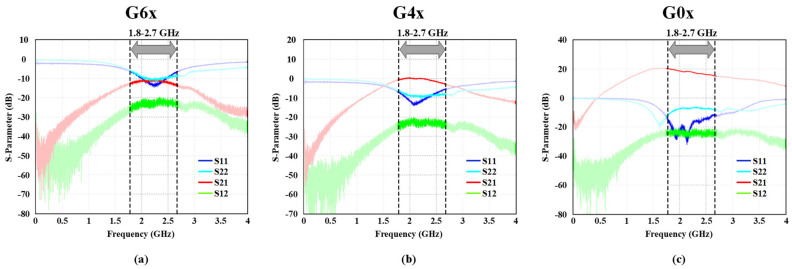
Measured S-parameter results in Mode0 for LB frequencies: (**a**) −11 dB of power gain step, (**b**) 0 dB of power gain step, and (**c**) 20.5 dB of power gain step.

The measured S-parameter results of power gain (S21), reverse isolation (S12), input matching (S11), and output matching (S22) of three sampled gain steps (G6x, G4x, and G0x) are demonstrated in Figure 9a–c. An 8.2 nH external inductor is employed to match the input impedance of the RF front-end in LB. Since ESD protection degrades S11 because of parasitic capacitors, the proposed LNA can satisfy the input matching specification, as illustrated in Figure 9a–c.

**Figure 10:**
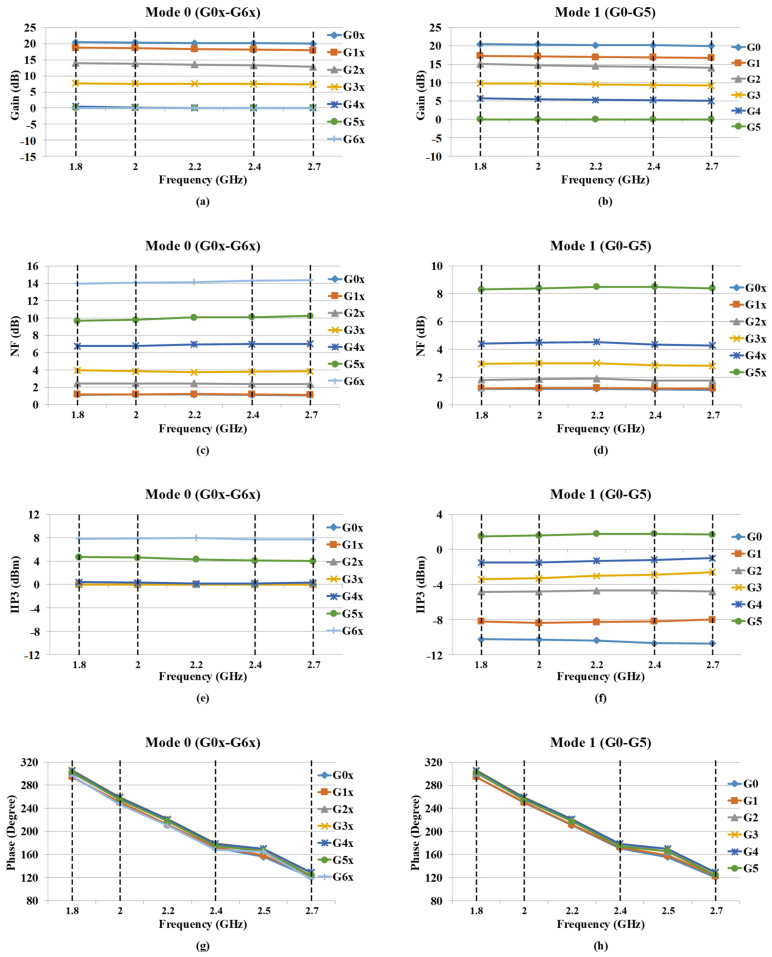
Measured performance graph summary of: (**a**) Mode0 power gain steps, (**b**) Mode1 power gain steps, (**c**) Mode0 NF, (**d**) Mode1 NF, (**e**) Mode0 IIP3, (**f**) Mode1 IIP3, (**g**) Mode0 output phase, (**h**) Mode1 output phase, in LB (1.8–2 GHz), MB (2–2.4 GHz), and HB (2.4–2.7 GHz), respectively.

Figure 10a–h indicates graphs of the measured RF performances over the entire input frequency range (1.8 GHz to 2.7 GHz) for all power gain steps in Mode0 and Mode1. As shown in Figure 10a and Figure 10b, the RF front-end provides a dynamic range of power gain from −11 dB to 20.5 dB (7 steps) and from −3 dB to 20.5 dB (5 steps) for Mode0 and Mode1, respectively.

Since the RF front-end is located in the first stage of an RX chain, the noise figure performance is one of the most important characteristics, as discussed in Section 3. The best value of 1.15 dB for NF performance is achieved in the highest power gain (G0x/G0). The graphs of NF performances for all gain steps and different frequency bands in Mode0 and Mode1 are stated in Figure 10c and Figure 10d, respectively. Since the SNR of the input signal is increased significantly in lower gain steps, the degradation of NF performance could be insignificant.

The IIP3 performance of all steps for Mode0 and Mode1 are graphed in Figure 10e,f, respectively. The IIP3 performance is improved from −10.75 dBm to 7.7 dBm and from −10.75 dBm to 1.5 dBm in Mode0 and Mode1, respectively, while the power gain decreases from the highest power gain to the lowest power gain.

Figure 10g,h illustrate the graphs of phase discontinuity performance over the entire input frequency range (1.8 GHz to 2.7 GHz) for all power gain steps in Mod0 and Mode1, respectively, where the difference between two subsequence power gain steps does not exceed 10 degrees.

The comparison table shows the performances of the proposed architecture with other structures. As mentioned in Table 7, the dynamic range of power gain, NF performance, and adaptive power consumption over a wide range of the input frequency from 1.8 GHz to 2.7 GHz is the superiority of this work over similar work, while ESD protection is employed. The power consumption of the proposed LNA is greater in the highest power gain in comparison with other works, nevertheless, the proposed RF front-end offers better NF and power gain performances. In comparison with [15], even though it is offering a lower power consumption and a better IIP3 but it is occupying a larger area that increases the fabrication cost in expensive CMOS-SOI processes, significantly. Nonetheless, the value of NF is higher than the proposed work in this paper. In terms of the number of gain steps, the proposed LNA provides 12 adaptive gain steps, which is more than in other work.

## 5. Conclusions

In this paper, a triple-band multi-gain RF front-end is designed and implemented for LTE mobile communication devices. The proposed architecture is fabricated in 65 nm SOI CMOS technology and experimental results indicate an overall dynamic range of 31.5 dB, while the highest power gain is obtained 20.5 dB. The proposed LNA shows a minimum NF of 1.15 dB over the wide bandwidth. IIP3s of −10.2 dBm and 8.6 dBm are obtained for the maximum and minimum power gain steps, respectively. Measurement results indicate that the proposed multi-core LNA consumes a gain-adaptive current consumption of 16.8 mA and 1.33 mA from an LDO (1 V) in the highest power gain and the lowest power gain steps, respectively. The power consumption changes according to power gain steps. The difference of the output phase does not exceed 10 degrees, as illustrated in the measurement results.

## Figures and Tables

**Table 1:** Programmable digital control bits of input-attenuator.

Gain Step	ATT<0>	ATT<1>	ATT<2>	ATT<3>
G0x/G0	ON	OFF	OFF	OFF
G1x/G1	ON	OFF	OFF	OFF
G2x/G2	OFF	ON	OFF	OFF
G3x/G3	OFF	ON	OFF	OFF
G4x/G4	OFF	OFF	ON	OFF
G5x/G5	OFF	OFF	OFF	ON
G6x	OFF	OFF	OFF	ON

**Table 2:** Programmable digital control bits of capacitor bank.

Freq. Band	CB<0>	CB<1>	CB<2>	CB<3>
LB	OFF	ON	OFF	ON
MB	ON	OFF	OFF	OFF
HB	OFF	OFF	ON	OFF

**Table 3:** Programmable digital control bits of the load switching and additional cascode devices.

Gain Step	AT<0>	AT<1>	AT<2>	AT<3>	AT<4>	AT<5>
G0x	OFF	OFF	OFF	OFF	OFF	OFF
G1x	OFF	ON	ON	OFF	OFF	OFF
G2x	ON	ON	ON	OFF	OFF	OFF
G3x	ON	ON	ON	ON	OFF	OFF
G4x	ON	ON	ON	OFF	ON	OFF
G5x	ON	ON	ON	OFF	OFF	ON
G6x	ON	ON	ON	OFF	OFF	ON
G0	OFF	OFF	OFF	OFF	OFF	OFF
G1	ON	ON	ON	OFF	OFF	OFF
G2	ON	ON	ON	OFF	OFF	OFF
G3	ON	ON	ON	OFF	OFF	OFF
G4	ON	ON	ON	OFF	OFF	OFF
G5	ON	ON	ON	OFF	OFF	ON

**Table 4:** Biasing voltage value for all gain steps.

Gain Step	VB1 (V)	VB2 (V)	VB3 (V)	VB4 (V)	VB5 (V)	VB6 (V)
G0x/G0	0.4	0.75	0.75	0	0	0
G1x/G1	0.34	0.75	0.75	0	0	0
G2x/G2	0.34	0.75	0	0	0	0
G3x/G3	0.34	0	0.75	0	0	0
G4x/G4	0	0	0	0.34	0.75	0
G5x/G5	0	0	0	0.34	0	0.75
G6x	0	0	0	0.34	0	0.75

**Table 5:** Transistor sizes of the proposed RF front-end.

Components	Parameter
MN1, MN2	W = 40 μm, L = 65 nm, m = 4
MN3, MN4	W = 40 μm, L = 65 nm, m = 2
MN5	W = 30 μm, L = 65 nm, m = 2
MN6	W = 36 μm, L = 65 nm, m = 1
MN7	W = 28 μm, L = 65 nm, m = 1
MN8	W = 22 μm, L = 65 nm, m = 1
MN9, MN10	W = 5 μm, L = 65 nm, m = 1
MN11	W = 2 μm, L = 65 nm, m = 1
MP1, MP2, MP3	W = 40 μm, L = 65 nm, m = 1

**Table 6:** Summary of power consumption for all gain steps.

Gain Step	G0x/G0	G1x/G1	G2x/G2	G3x/G3	G4x/G4	G5x/G5	G6
Power consumption (mW)	16.8	10.2	7.06	3.51	2.7	1.42	1.33

**Table 7:** Performance summary and comparison to conventional structures.

	This Work	[15]	[16]	[17]	[18]	[19]	[20]
Freq. (GHz)	1.8–2.7	4–11	5.9	0.6–3.15	0.3–3.5	0.1–3.4	0.02–4.5
Power Gain (dB)	−11–20.5 (12-gain)	21	9	20.2	14.6	18.2	11.2–20.4
S11 (dB)	<−10	<−10	−11	N/A	<−10	N/A	N/A
NF (dB)	1.15 @ HG	2.75	1.34	<3.1	2.9–3.5	3.4	3.2–5.4
IIP3 (dBm)	−10.75 @ HG	6.5	N/A	−2.1	1.2–4.7	−1.46	−8
Power (mW)	16.8 @ HG	5.15	9.6	6	14.8	3.3	15.6
ESD (kV)	2	N/A	N/A	N/A	N/A	N/A	N/A
$\mathrm{Size}\;({\mathrm{mm}}^{2}$)	0.308	1.32	0.86	0.026	0.2754	0.72	0.62
Tech. (nm)	65	65	130	180	180	130	28
